# Geologically-constrained GANomaly network for mineral prospectivity mapping through frequency domain training data

**DOI:** 10.1038/s41598-024-56644-8

**Published:** 2024-03-14

**Authors:** Hamid Sabbaghi, Seyed Hassan Tabatabaei, Nader Fathianpour

**Affiliations:** Department of Mining Engineering, Isfahan University of Technology, Isfahan, Iran

**Keywords:** Generative adversarial network, Geologically-constrained GANomaly, Multi-element geochemical anomaly, Mineral prospectivity mapping, Au–Cu mineralization, Frequency domain data, Environmental sciences, Solid Earth sciences, Chemistry, Engineering, Mathematics and computing

## Abstract

Generative adversarial networks (GAN) and various deep autoencoders have been frequently executed to recognize multi-element geochemical anomalies linked to different ore resources in recent decade. Efficient recognition of multi-element geochemical anomaly patterns is a significant issue in mineral exploration targeting. Traditional procedures have not sufficient capability to perform efficient pattern recognition. While, deep learning algorithms as influential subset of machine learning algorithms can present magnificent conclusions in classification and pattern recognition. Because those have robust ability in extracting high-level features of complex inputs. Although, many deep learning algorithms were used to recognize geochemical anomalies but the GANs have demonstrated specific dignity in recognizing multi-element geochemical anomaly patterns. But, these frameworks should be constrained to learn geological knowledge and yield reasonable potential maps. In this regard, a novel geologically-constrained GANomaly was trained with frequency domain training data to recognize multi-element geochemical anomalies. Application of the geologically-constrained GANomaly network with considering mineral system parameters of the Au–Cu mineralization in the Feyzabad district, NE Iran was eventuated to suitable results. The success-rate curves demonstrated that produced map of frequency domain geochemical data has traced 86.68% Au–Cu occurrences via 30% corresponded area while produced map of spatial domain geochemical data has traced 80.13% Au–Cu occurrences via 30% corresponded area.

## Introduction

Satisfactory recognition of multi-element geochemical anomalies related to mineralization is an important issue for mineral exploration targeting in regional scale^1^. Because, recognition of multi-element geochemical anomalies in regional scale is generally performed applying stream sediments geochemical data^2^. Stream sediments geochemical data is mostly influenced through complexity of geological features^2,3^. Therefore, stream sediments geochemical data is a complex multivariate input for processing frameworks. Although, shallow learning algorithms have not sufficient ability to process this complex data^4,5^. But, deep learning (DL) algorithms have repeatedly demonstrated their capability in processing complex data^2,3,6–8^. Among various DL frameworks, generative adversarial networks (GAN), deep autoencoders and deep convolutional networks have proved their specific dignity in mineral prospectivity mapping (MPM) and geochemical anomaly detection^6,7,9–11^. Before, GANs were mostly being applied to process remote sensing or seismic data. While, these frameworks have been recently executed to recognize geochemical anomaly patterns linked to mineralization^6,7^. A GAN is derived from the zero-sum game in game theory, which enables discriminator and generator to improve the performance of the model during a mutual game. The Wasserstein, convolutional, conditional, cycle and semi-supervised are known variations of the GAN which have been employed for geochemical anomaly detection, style transfer, image recognition or image generation^11^. Also, various deep autoencoders and deep convolutional networks were widely employed to recognize geochemical anomaly patterns related to different mineralizations due to popularity and great ability in feature extraction^12^. Accordingly, the GANomaly framework as a combination of the deep convolutional autoencoder (DCAE) and GAN is considered as a vigorous procedure for geochemical anomaly detection. It is noteworthy, application of the robust DL approaches such GANomaly as purely data-driven way can not be eventuated to reliable results^6,13^. Because, purely data-driven DL frequently ignore expert and domain knowledge, leading to difficulty in interpretability from a geological perspective. Hence, importing mineral system parameters as geologically constraints within the DL structures is a regardable idea for improving their inference power^6,14^. Constructing geological constraint of ore-controlling feature allows the model designed to learn geological knowledge and yields reasonable potential maps which rarely include user bias problem. Also, other proposed improvement idea is training DL algorithms with frequency domain (FD) geochemical data. The FD geochemical data contains rather exploratory information than spatial domain (SD) geochemical data^15^. Filtered data (FD geochemical data) is smoother and cleaner than the original data (SD geochemical data). Because, corresponding filter is designed according to the frequency of different geochemical data noise, which is applied to filter out the noise in the geochemical data. In fact, the FD geochemical data comprises superlative information related to mineralization occurrences that is not posed applying the SD geochemical data. Recent novel contributions have mostly attempted to overcome user bias problem in MPM^16^. We intend to consider application efficacy of the FD training data whether can decrease this problem. This research applies FD geochemical layers to train a novel geologically-constrained GANomaly for MPM. The success-rate curves demonstrate that produced FD geochemical map has rather consistency to the Au–Cu occurrences in the Feyzabad district, NE Iran. The FD geochemical map has predicted more proportion of mineralization occurrences within less proportion of the corresponding study area. Because, FD geochemical map rarely includes user bias problem.

## Region of interest

**Figure 1:**
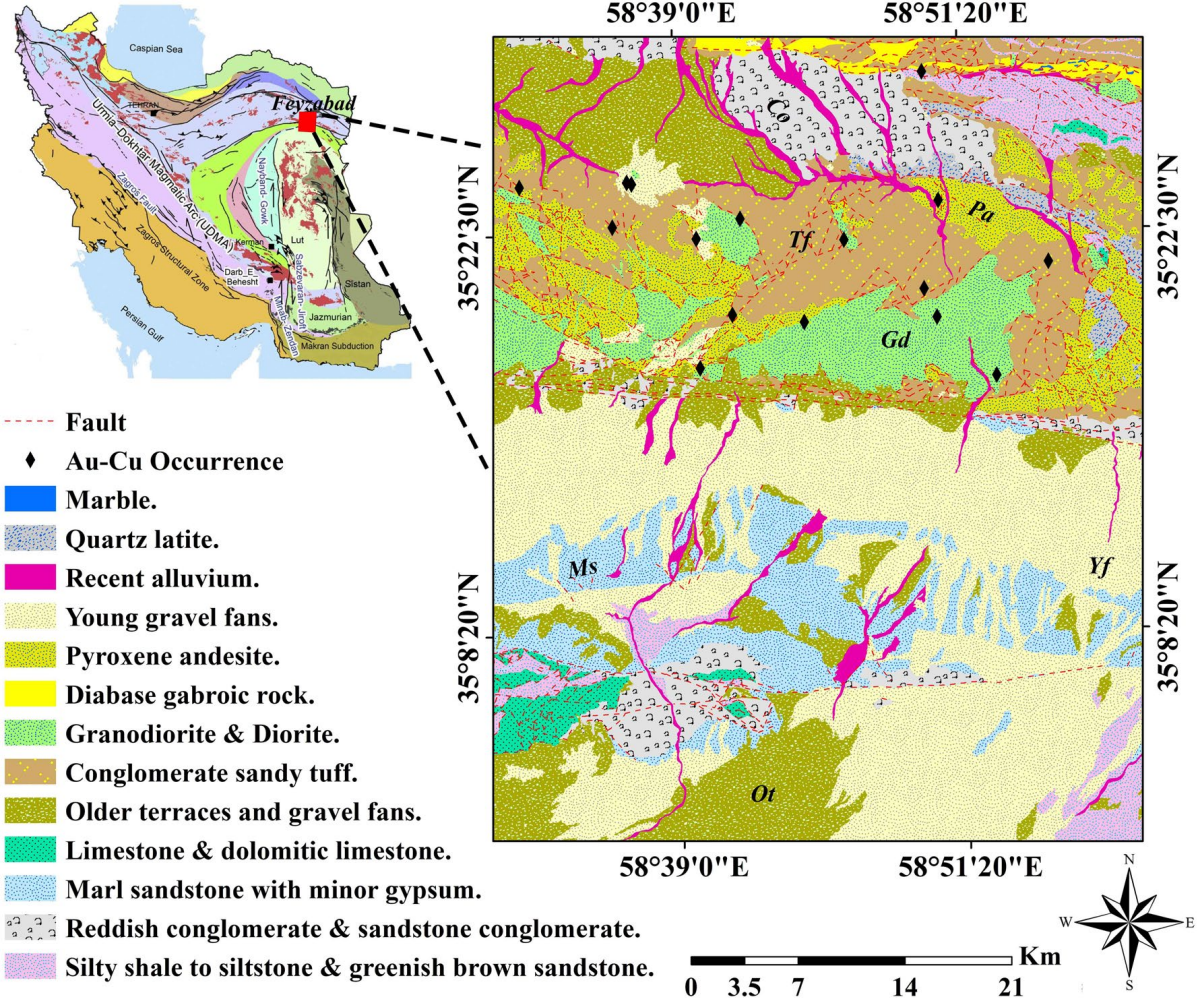
Simplified geological map (1:100,000) of the Feyzabad district.

A main mineral potential zone from NE Iran is the Feyzabad district. The Feyzabad is known as a high potential area of the iron oxide copper–gold (IOCG) and vein-type Au–Cu mineralizations which is restricted between 58° 30′ 0″ E and 59° 0′ 0″ E longitudes and 35° 0′ 0″ N and 35° 30′ 0″ N latitudes. This area is a segment of the boundaries of the internal Iranian microcontinent which places between the Loot Block and the Central Iran zones. It is seen, numerous faults and fractures are related to Au–Cu mineralization occurrences in this area. In this regard, the darouneh fault as the longest fracture plays a significant role in forming Au–Cu deposits of the Feyzabad district. Granodiorite, diorite, pyroxene andesite and diabase gabbroic rock are the most significant volcanic structures which are frequently observed there (Fig. 1). Also, alternations of sedimentary and carbonated rock units comprising reddish and sandstone conglomerate, gypsiferous marl, dolomitic limestone, silty shale and quartz latite which belong to middle- to upper-Cambrian era accompany mentioned volcanic rock units (Fig. 1). The vein-type Au–Cu and the IOCG deposits are mainly hosted by diorite and granodiorite intrusions of Eocene–Oligocene age in this area^17^. Appropriate pathfinder elements Au, Cu, Sb, Zn and Pb were chosen to trace Au–Cu mineralization occurrences in the study area^3,18^.

## Methods

### Insight of constructing constraint

Three subsystems containing pre-mineralization, syn-mineralization and post-mineralization are the most significant systems related to different mineralizations. In this issue, considering mineral system parameters such (1) source and composition of the forming fluids, (2) crustal structure and tectonic history, (3) fluids pathways, (4) mechanism of concentrated fluids flow and (5) necessary mechanisms for depositing Au–Cu such chemical and physical barriers can avoid to construct purely data-driven models^18^. These critical ore-forming processes are not mappable but those should be translated to augment DL models applied. Accordingly, a hierarchical procedure as converting data to information, information to knowledge and knowledge to insight for translating critical ore-forming processes should be performed. An example of the converting data to information is discovering correlations of the pathfinder elements of mineralization. Also, understanding mineralization type based on identified pathfinder elements is considered as an example of converting information to knowledge. Eventually, combining geochemical knowledge with geological knowledge is eventuated to insight of constructing constraints and credible mapping.

### Transforming data domain and filtering

Transforming domain of geochemical data is performed to access newer information. Frequencies domain of geochemical data can reveal more hidden characteristics than spatial geochemical data through implementing two-dimensional Fourier transform (2DFT)^15,19^. The 2DFT can be expressed as follow:

1 $$F\left( {K_{x} {, }K_{y} } \right) \, = \mathop \smallint \limits_{ - \infty }^{{\infty { }}} \mathop \smallint \limits_{ - \infty }^{\infty } f\left( {x,y} \right)\cos \left( {K_{x} x + K_{y} y} \right)dxdy - i\mathop \smallint \limits_{ - \infty }^{\infty } \mathop \smallint \limits_{ - \infty }^{\infty } f\left( {x,y} \right)\sin \left( {K_{x} x + K_{y} y} \right)dxdy$$

where $f(x, y)$, ${K}_{y}$ and ${K}_{x}$ are considered as spatial domain data, wave numbers with respect to the *y* and *x* axises. Wave numbers are proportionally increased as follow:

2 $$\lambda \, = { 2}\pi \sqrt {\left( {\frac{1}{{K_{x}^{2} }} + \frac{1}{{K_{y}^{2} }}} \right)} \;\;\;\;\lambda_{x} = 2\pi /K_{x} \;{\text{and}}\;\lambda_{y} = 2\pi /K_{y}$$

Hence, a surface multi-element geochemical map which is considered as a function *f(x, y)* in the spatial domain, can be converted into *F(K*_*x*_*, K*_*y*_*)* which* I(K*_*x*_*, K*_*y*_*)* and* R(K*_*x*_*, K*_*y*_*)* are its imaginary and real parts, respectively. Accordingly, its power spectrum can be calculated as:

3 $$E\left( {K_{x} , \, K_{y} } \right) = R^{2} (K_{x} , \, K_{y} ) + I^{2} (K_{x} , \, K_{y} )$$

Decreasing noise of the transformed data through filtering procedures is a common operation to process frequency domain geochemical data. The *I(K*_*x*_*, K*_*y*_*)* and* R(K*_*x*_*, K*_*y*_*)* can be achieved multiplying filter function *G(K*_*x*_*, K*_*y*_*)* and removing or boosting several wave number ranges. Filters are generally performed according to wave numbers and not power spectrum values^15^. One of the most popular and applicable filters is Butterworth filter which was initially introduced by Stephen Butterworth in 1930. This filter has been discussed as low-pass for denoising various transformed data in recent years^20–22^. Its formula can be expressed as follow:

4 $${\left|{f}_{l}(w)\right|}^{2}= \frac{1}{1+({\frac{w}{{w}_{c}})}^{2n}}$$

where *w*_*c*_ and *n* are cutoff frequency and order of filter, respectively. Filtered data via applying Butterworth filter are more smooth and cleaner than primitive data.

### The GANomaly framework

**Figure 2:**
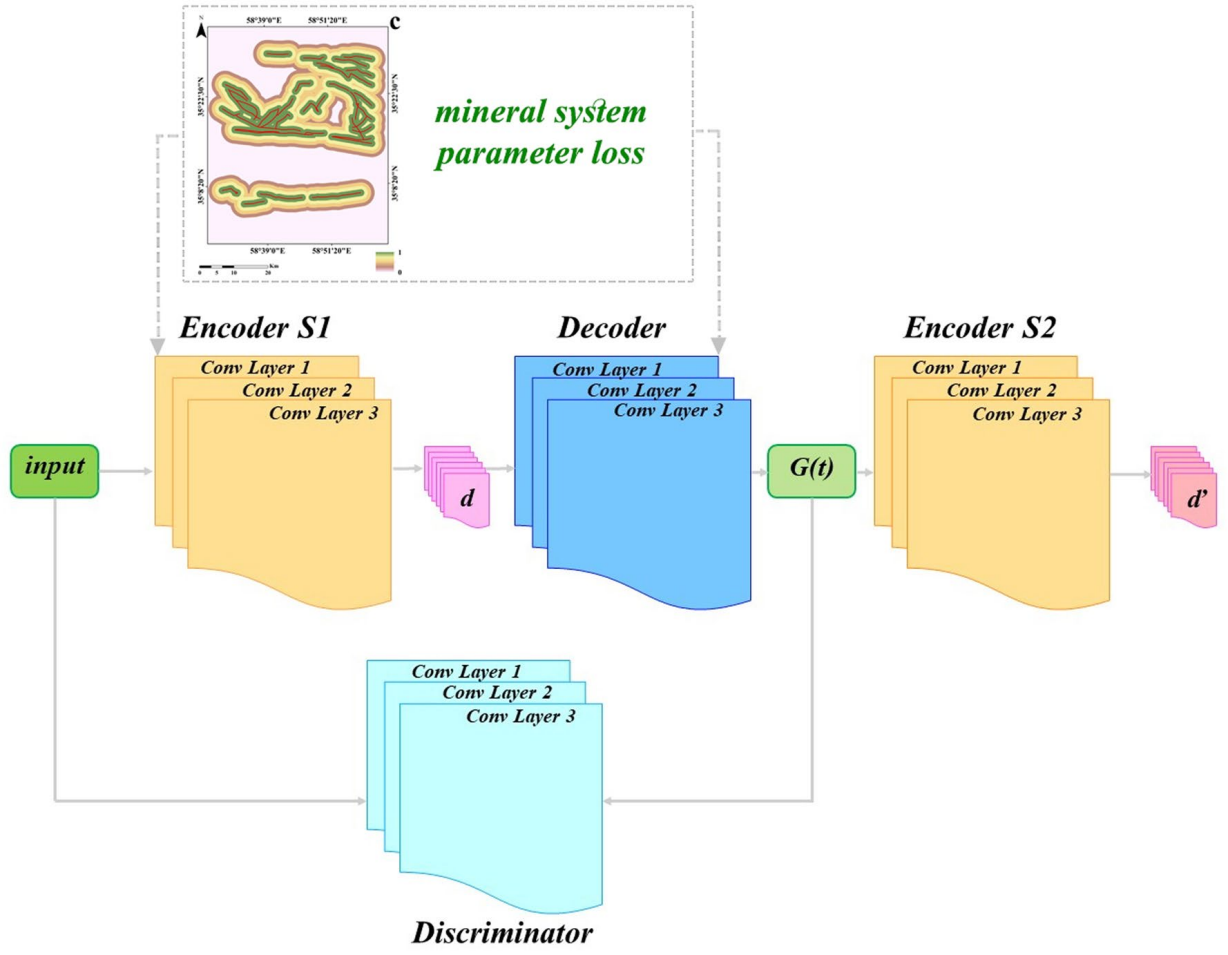
A diagram depicting structure and processing layers of the GANomaly.

Fundamental idea of the GAN has originated of the game theory. Indeed, based on a mutual game, its two main sections comprising generator and discriminator are trained to improve framework performance. In comparison to other DL approaches, GANs can increase quality of samples produced and achieve information of latent space in generative procedure without wasting sampling speed. As an augmented GAN, the GANomaly framework was initially carried out by^23^. Then, this framework was developmentally applied to recognize geochemical anomalies by^11^. The GANomaly structure includes a generator section with random noise input and new generated samples as output. Discriminator section of the GANomaly as a classifier is adversarially obliged to discriminate fake generated samples of the real samples. This adversarially procedure continues until fake generated samples of generator section be plausible for discriminator section and be not recognizable than the real samples^24^. Latent vector space and original data space can be trained to the GANomaly through hybridization of a GAN structure with the DCAE. In fact, GANomaly can improve recovering ability of decoder applying adversarial procedure in comparison to traditional autoencoders^11^. The GANomaly is contained three sub-sections (Fig. 2). The first part (encoder S1 and its decoder) presents input *t* to encoder *S1* for achieving the latent feature vector *d* and its decoder perfects reconstruction procedure via applying latent feature vector *d*. The second part presents reconstructed information *ť* to encoder *S2* for achieving latent feature vector *ď*. Consistency of the *S1* and *S2* is confirmed via same dimensions of *d* and *ď*. The third part as a discriminator is adversarially obliged to discriminate *ť* from *t*. When the discriminator has difficulty in discriminating between these, it reveals that the generated data are extremely similar to the normal sample data at this time. When abnormal samples are input, the encoder *S1* encodes the abnormal samples to achieve the latent feature vector *d*, but the decoder cannot reconstruct the abnormal samples correctly. Because the training phase only uses normal samples for modeling, and its parameters are not appropriate for abnormal samples. Therefore, the latent feature vector *d’* obtained by encoder *S2* is extremely different from the latent feature vector *d*, and abnormal samples are recognized detecting the difference between these two vectors. In this research, each three sub-sections have been constructed employing the DCAE. Geologically-constrained GANomaly is contained three loss functions for sub-sections and a loss function for geologically constraint defined. The first loss function of the GANomaly can enhance decoder power in restoring features and stability of trained model. The generator (*G*) of the GANomaly should be updated according to internal representation of discriminator. In fact, *t* as an input of supposed function *f(‧)* has been collected of the distribution *P*_*t*_. Also, conclusion of the middle layer feature of the discriminator (*D*) is output of the *f(‧).* Differences between the fake generated features and real data features can be considered following adversarial loss function:

5 $${O}_{adv}= {E}_{t\sim Pt}{\Vert f\left(t\right)- {E}_{t\sim Pt}f(G\left(t\right))\Vert }_{2}$$

The GANomaly framework regulates generator for optimizing similarity between real samples and fake generated samples. This procedure is performed through loss function *O*_*con*_ for calculating distances between the fake generated samples *ť* and real samples *t*.

6 $${O}_{con}= {E}_{t\sim Pt}{\Vert t- G(t)\Vert }_{1}$$

Eventually, distance between the latent feature vector *ď* and latent feature vector *d* is minimized defining the third loss function of the GANomaly. This loss function obliges generator to learn how to encode characters of the fake generated samples based on normal samples.

7 $${O}_{enc}= {E}_{t\sim Pt}{\Vert {G}_{E}(t)- E(G\left(t\right))\Vert }_{2}$$

When training of the model was completed, the testing sample achieves latent feature vector *d* applying part *S1* and then achieves reconstructed latent feature vector *ď* applying part *S2*. Distinguishing abnormal samples of the testing data can be performed through average absolute error *H(t)* between reconstructed latent feature vector *ď* and latent feature vector *d* as follow:

8 $$H(t) =\frac{1}{m}({\Vert {G}_{E}\left(t\right)-E\left(G\left(t\right)\right)\Vert }_{1})$$

### Constructing constraint of ore-forming features

A geologically constraint as a nonlinear correlation between the Au–Cu mineralization occurrences and controlling features such buffered fault layer was employed for this research. This constraint can be expressed as follow:

9 $$\rho = C{d}^{\alpha -3}$$

where *C*, *α*, *ρ* and *d* are a constant value, multifractal singularity index, density of the Au–Cu mineralization occurrences, distance between the Au–Cu mineralization occurrences and geological controlling features, respectively. While *α* be less than 3, there is an important spatial correlation between geological controlling features and locations of the Au–Cu mineralization occurrences^14^. In fact, this constraint as a knowledge factor according to mineral system parameter was applied to improve objective function of the GANomaly framework (Fig. 2). Detailed description of constructing geological constraint is accessible in^10,14^. Accordingly, geological knowledge loss function is calculated as follow:

10 $${O}_{pro}= \frac{1}{N} \sum_{i=1}^{N}{\Vert Lt- {\omega }_{pro} \Vert }_{2}$$

where *Lt* is predictive layer and ${\omega }_{pro}$ is weights of a geological controlling feature which is computed as follow:

11 $${\omega }_{pro}= \frac{\rho }{max(\rho )}= \frac{ C{d}^{\alpha -3}}{max(\rho )}$$

Accordingly, objective function (total loss function) of the geologically-constrained GANomaly framework is presented as follow:

12 $${O}_{t}= {\omega }_{adv}{O}_{adv}+{\omega }_{con}{O}_{con}+{\omega }_{enc}{O}_{enc}+ {{\omega }_{pro}O}_{pro}$$

where *ω*_*enc*_, *ω*_*con*_ and *ω*_*adv*_ are regulable weight parameters of the GANomaly loss functions. Noise interferes with the reconstruction error of the sample which can affect the recognition ability. The GANomaly network no longer uses the reconstruction error of the sample as the foundation for anomaly recognition during the detection phase; instead, reconstruction errors of the deeper latent vector is applied for anomaly recognition. Therefore, reconstruction error of the deeper latent vector can be considered assigning regulable weight parameters to loss functions. Also, controlling balance between the GANomaly loss functions and loss function of mineral system parameter is performed defining ${\omega }_{pro}$.

### Geochemical sample preparation and analysis

The study area has dimensions of 44 × 54 km^2^ which a dense sampling grid (1.4 × 1.4 km^2^) has been performed there. Stream sediments samples (1033) were collected to check changing rate of concentrations of 27 elements across the Feyzabad district. Collected geochemical samples were analyzed using a combined inductively coupled plasma-optic emission spectroscopy and mass spectroscopy (ICP-OES/ICP-MS) after a near-total 4-acid digestion (hydrochloric, nitric, perchloric, and hydrofluoric acids)^25^. Also, analyzing precision (< 10%) was measured applying duplicated sub-samples for each 20 measurements.

## Results and discussion

### Transforming geochemical data and preparing predictive layers

**Figure 3:**
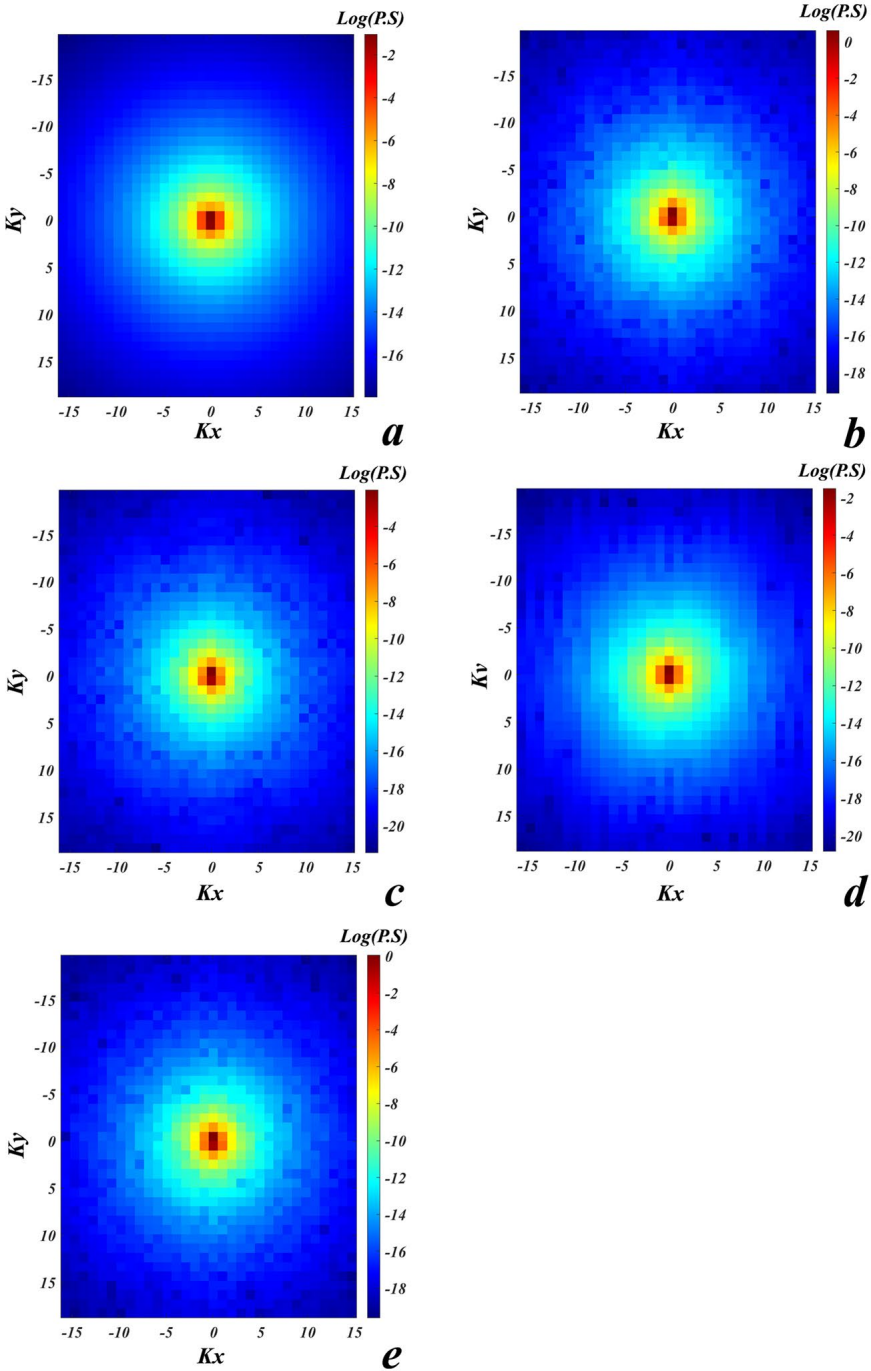
Spectrum values of the pathfinder elements, (**a**) Au, (**b**) Cu, (**c**) Sb, (**d**) Zn and (**e**) Pb.

**Figure 4:**
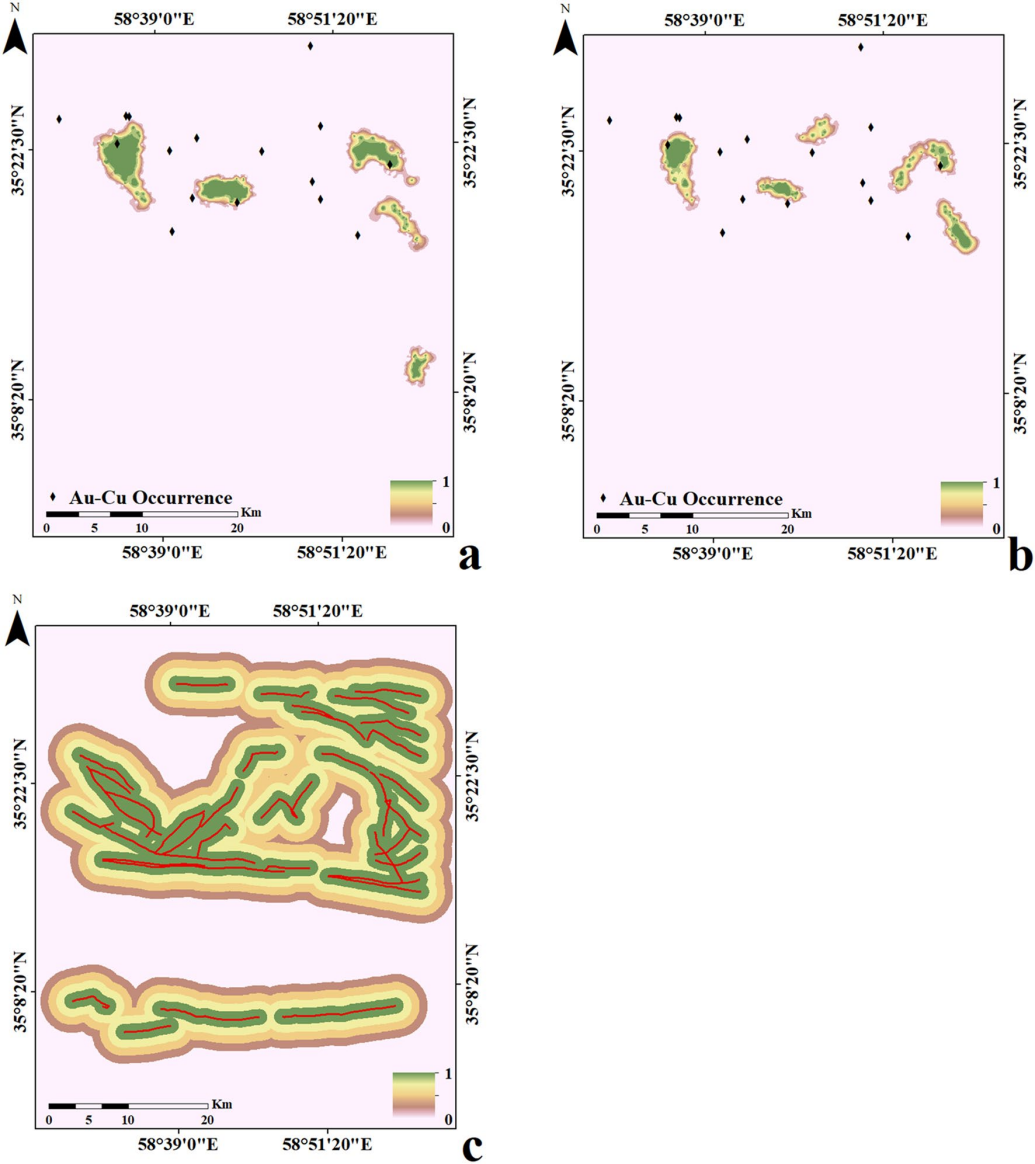
Several predictive layers for training GANomaly framework, (**a**) SD geochemical layer of Au, (**b**) FD geochemical layer of Au and (**c**) Fault predictive layer as geological constraint.

Stream sediments geochemical data includes inherent closure problem^26^. Hence, the centered log-ratio (clr) transformation was performed to eliminate data closure problem using Eq. (13).

13 $$clr (x) = \left({\text{log}}\left(\frac{{x}_{1}}{g\left(x\right)}\right),\dots ,({\text{log}}\frac{{x}_{D}}{g(x)})\right)$$

where *x*, *x*_*D*_ and *g(x)* are vector of the composition with *D* dimensions, Euclidean distances between distinct variables and geometric mean of the composition *x* respectively^27^. Then, the 2DFT was performed. The FD data is comprised power spectrum values and the wave numbers in *x* and *y* axises. The power spectrum *E(Kx, Ky)* values calculated for pathfinder elements Au, Cu, Sb, Zn and Pb have been depicted in Fig. 3. The values which are closed to the center of these plots have low wave numbers and frequencies. These values are high power spectrum values which decrease moving away from the plot center (Fig. 3). The low and high wave numbers present low- and high-frequency values of concentrations in geochemical data. Denoising power spectrum values was performed through Butterworth filter and then the FD data was inverted to produce the FD layers of geochemical elements. The SD and FD geochemical data of the pathfinder elements were applied to produce predictive layers via executing inverse distance weighted (IDW) method with a grid of size 200 × 200 m^2^. As an example, the SD and FD predictive layers of element Au have been presented in Fig. 4a,b. Accordingly, five SD predictive layers and five FD predictive layers of the pathfinder elements Au, Cu, Sb, Zn and Pb were employed as input to train the GANomaly framework for tracing Au–Cu mineralization occurrences. The fault predictive layer with 4-ring buffered areas (with an interval of 1 km) was constructed as a geological constraint based on mineral system parameter to improve loss function of the GANomaly framework (Fig. 4c). This predictive layer is guidance and restriction factor for the designed model due to regard mineral system parameter which is eventuated to reliable exploration targeting.

### Mineral prospectivity mapping and validation

**Figure 5:**
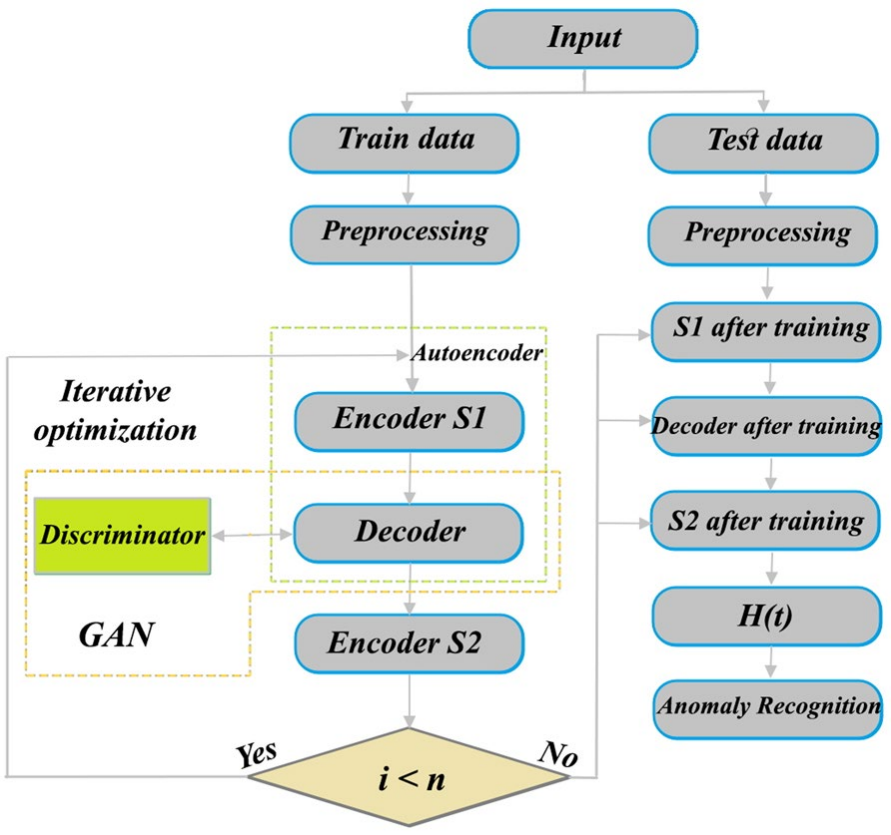
A diagram exhibiting extraction process of information applying the GANomaly.

**Figure 6:**
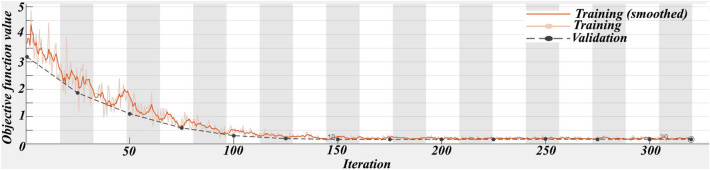
Decreasing compositional loss function value in total iterations.

**Figure 7:**
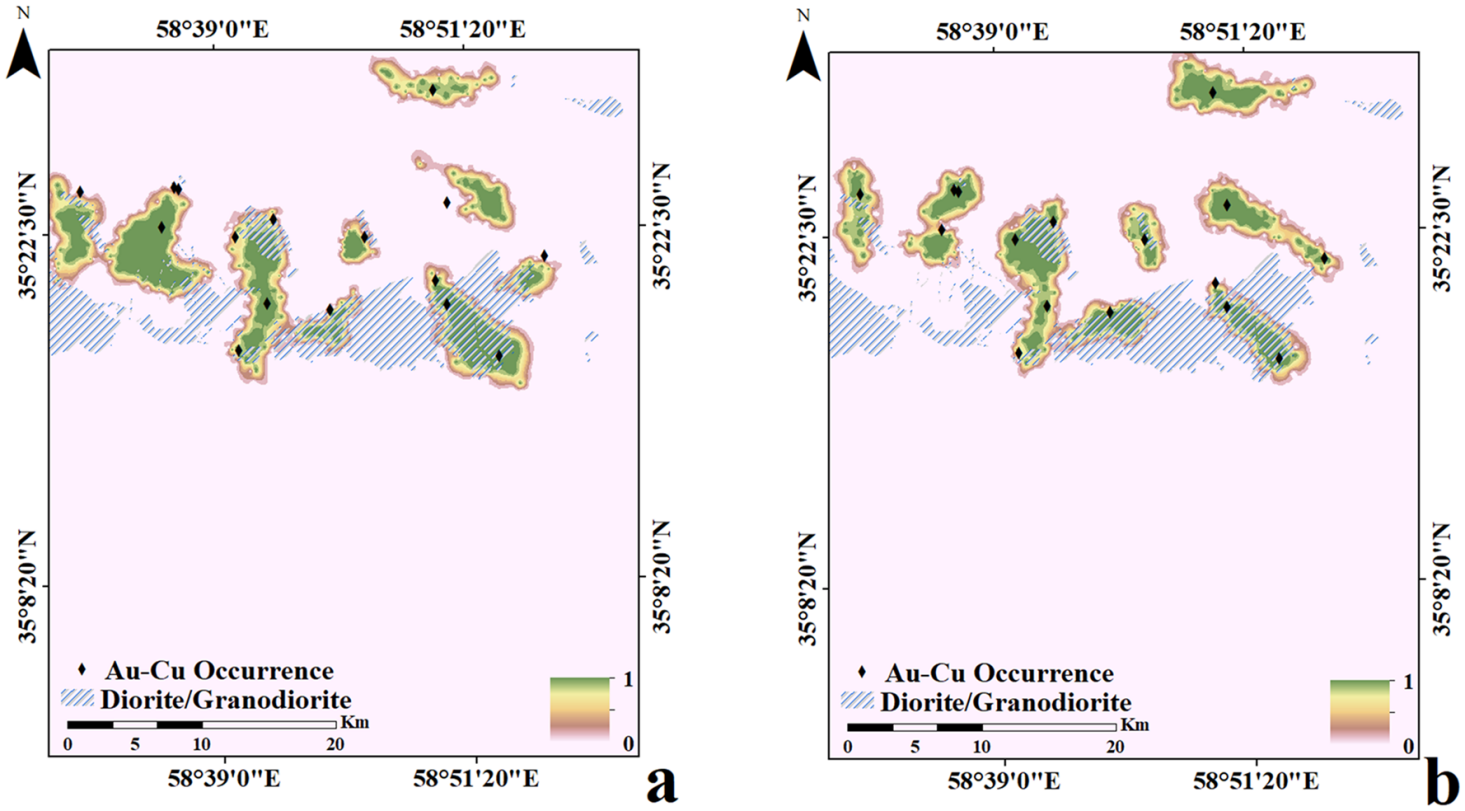
Obtained multi-element geochemical anomaly maps applying geologically-constrained GANomaly with, (**a**) the SD training data and (**b**) the FD training data.

**Figure 8:**
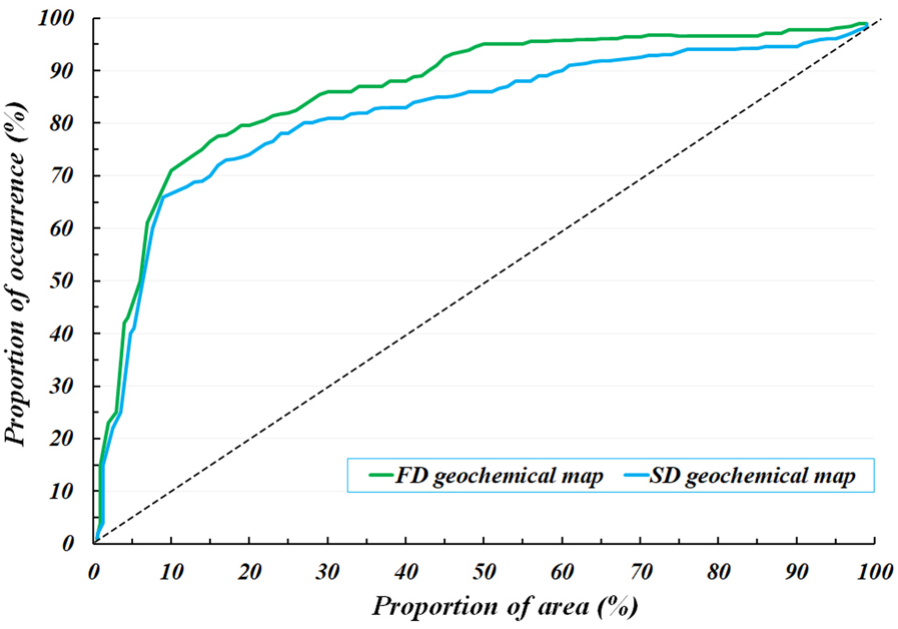
Success-rate curve of the SD geochemical anomaly map versus success-rate curve of the FD geochemical anomaly map.

Each five same predictive layers (FD or SD) were combined into a set of input feature vectors at each cell location in the set of grids. All cells of the same predictive layers were divided as training data (30%) and testing data (70%) and were applied to trace Au–Cu mineralization occurrences in the Feyzabad district. The MATLAB R2022a environment was applied to implement the geologically-constrained GANomaly framework. In encoder S1, convolutional layers had 64, 128 and 256 kernels respectively. Also, deconvolutional layers had 256, 128 and 64 kernels in decoder part respectively. Kernel size of decoder and encoder were also fixed as 4 × 4. Optimizing the output distribution and training efficiency were improved implementing LeakyReLU activation function and Batch normalization in intermediate convolution layer. The batch size and initial learning rate were experimentally fixed to 128 and 0.0001 respectively. Also, Adam optimization algorithm was applied to optimize the objective function of the framework designed. A schematic diagram of the GANomaly sections with extraction process of information has been displayed in Fig. 5. Based on Eq. (8), samples with a high difference values of the latent feature are regarded as geochemical abnormal points (Fig. 5). Augmented loss function via geological constraint constructed of ore-controlling feature allows the model designed to learn geological knowledge and yields reasonable potential maps which rarely include user bias problem. Defining geologically-constrained loss function is caused that abnormal areas detected be more consistent with known geological knowledge than unconstrained loss function of network and enabled a more accurate delineation of abnormal regions. Indeed, the proposed model as a novel DL black-box can appropriately consider the spatial distribution of mineral deposits and improve the interpretability and generalization of geochemical pattern recognition. Optimization of the objective function values in whole iterations of training geologically-constrained GANomaly framework has been presented in Fig. 6. Continuously decreasing of objective function value can demonstrate a well-trained framework for this research. Objective function has been eventually converged to 0.17 at a steady state since iteration 200th. Produced multi-element geochemical map of the SD data can be compared to produced multi-element geochemical map of the FD data through Fig. 7. Although, both obtained maps have consistency to the Au–Cu occurrences in the study area but the SD geochemical map displays lower success-rate for tracing the Au–Cu occurrences (Fig. 7a) than the FD geochemical map (Fig. 7b). Because, the FD geochemical data is contained more exploratory information. The success-rate curves can consider matching degree between detected mineralization occurrences and mineral potential zones. Accordingly, we applied success-rate curves to compare ability of both produced geochemical maps in tracing the Au–Cu occurrences (Fig. 8). The success-rate curve of the FD geochemical map demonstrates that 86.68% of the Au–Cu occurrences have been delineated through 30% corresponding study area. While, success-rate curve of the SD geochemical map has been plotted 80.13% of the Au–Cu occurrences through 30% corresponding study area. The greater prediction ability of the FD geochemical map confirms that filtered data has access to more exploratory information. In fact, training GANomaly framework with FD geochemical data has been eventuated to more consistent geochemical map. In addition, real differences of the FD geochemical data can be revealed employing augmented DL models.

## Conclusion

In this research, a geologically-constrained GANomaly was constructed to detect multi-element geochemical anomalies through regarding ore-forming processes. Application of this framework for detecting multi-element geochemical anomalies linked to the Au–Cu mineralization in the Feyzabad district from NE Iran, was successful with a great consistency to mineralization occurrences. Therefore, following conclusion remarks can be presented:

- Purely data-driven deep learning network requires to costraints for eventuating to reliable mineral exploration targeting.
- Mineral system parameters as constraints can reinforce deep learning algorithms to produce credible mineral potential maps.
- Frequency domain geochemical data includes rather exploratory information than spatial domain geochemical data because filtered data is cleaner and more smooth.
- A geologically-constrained deep learning model trained with frequency domain geochemical data can produce rather consistent potential maps to mineralization occurrences.
- Accordingly, a reinforced deep learning algorithm via mineral system parameters with suitable filtering can be a reliable procedure for decreasing user bias problem in mineral prospectivity mapping.

## Data Availability

The datasets used during the current study available from the corresponding author on reasonable request.
